# Diagnostic value of PET/CT with ^11^C-methionine (MET) and ^18^F-fluorothymidine (FLT) in newly diagnosed glioma based on the 2016 WHO classification

**DOI:** 10.1186/s13550-020-00633-1

**Published:** 2020-05-07

**Authors:** Tomoya Ogawa, Nobuyuki Kawai, Keisuke Miyake, Aya Shinomiya, Yuka Yamamoto, Yoshihiro Nishiyama, Takashi Tamiya

**Affiliations:** 1 Department of Neurological Surgery, Kagawa University, Faculty of Medicine, Miki-cho, Kagawa Japan; 2 Department of Neurological Surgery, Kagawa General Rehabilitation Hospital, 1114 Tamura-cho, Takamatsu-shi, Kagawa 761-8057 Japan; 3 Department of Radiology, Faculty of Medicine, Kagawa University, Miki-cho, Kagawa Japan

**Keywords:** ^11^C-methionine, ^18^F-fluorothymidine, Glioma, *IDH1* mutation, PET/CT

## Abstract

**Background:**

The molecular features of *isocitrate dehydrogenase* (*IDH*) mutation and chromosome 1p and 19q (1p/19q) codeletion status have pivotal role for differentiating gliomas and have been integrated in the World Health Organization (WHO) classification in 2016. Positron emission tomography (PET) with 3′-deoxy-3′-[^18^F]fluorothymidine (FLT) has been used to evaluate tumour grade and proliferative activity and compared with l-[methyl-^11^C]-methionine (MET) in glioma patients. Herein, we evaluated tracer uptakes of MET-PET/CT and FLT-PET/CT for differentiating glioma based on the 2016 WHO classification especially in relation to *IDH1* mutation status.

**Methods:**

In total, 81 patients with newly diagnosed supratentorial glioma were enrolled in this study. They underwent PET/CT studies with MET and FLT before surgery. The molecular features and histopathological diagnosis based on the 2016 WHO classification were determined using surgical specimens. The ratios of the maximum standardized uptake value (SUV) of the tumours to the mean SUV of the contralateral cortex (T/N ratios) were calculated on MET-PET/CT and FLT-PET/CT images.

**Results:**

The mean T/N ratios of MET-PET/CT and FLT-PET/CT in *IDH1*-wildtype tumours were significantly higher than those in *IDH1*-mutant tumours (*P* < 0.001 and *P* < 0.001, respectively). Receiver operating characteristic analysis for differentiating *IDH1* mutation status showed that the area under the curve of the FLT T/N ratio was significantly larger than that of the MET T/N ratio (*P* < 0.01). The mean T/N ratio of FLT-PET/CT in *IDH1*-wildtype tumours was significantly higher than that in *IDH1*-mutant tumours among grade II and III gliomas (*P* = 0.005), but this was not the case for MET-PET/CT. Both MET-PET/CT and FLT-PET/CT were able to distinguish between grade II and III gliomas in *IDH1*-mutant tumours (*P* = 0.002 and *P* < 0.001, respectively), but only FLT-PET/CT was able to distinguish between grade III and IV gliomas in *IDH1*-wildtype tumours (*P* = 0.029).

**Conclusion:**

This study showed that FLT-PET/CT can be used to determine the *IDH1* mutation status and evaluate glioma grade more accurately than MET-PET/CT. FLT-PET/CT can improve glioma differentiation based on the 2016 WHO classification, but caution must be paid for tumours without contrast enhancement and further studies should be conducted with more cases.

## Introduction

The updated 2016 edition of the World Health Organization (WHO) Classification of Tumours of the Central Nervous System (CNS) uses molecular parameters and histology to define the main tumours categories for the first time [1]. This represents a shift from the traditional way of using neuropathological diagnoses primarily based on microscopic features, to using biologically oriented diagnoses. Among several molecular features, *isocitrate dehydrogenase* (*IDH*) mutation and chromosome 1p and 19q (1p/19q) codeletion status are the genetic alterations that have had a significant impact on the new tumour classification [2]. Several studies have shown that *IDH1* mutation is a strong prognostic maker, and this may well be the most upstream genetic event in the tumourigenesis and may drive other genetic changes in tumour cells [3, 4].

Positron emission tomography (PET) with l-[methyl-^11^C]-methionine (MET) has been widely used as an imaging tool for brain tumour detection, tumour grading and prediction of prognosis in patients with gliomas [5]. Recent studies have examined MET uptake for differentiating glioma based on the 2016 WHO classification especially in relation to *IDH1* mutation and 1p/19q codeletion status. In general, MET uptakes in *IDH1*-wildtype gliomas were significantly higher compared with those in *IDH1*-mutant gliomas [6–8]. However, several amino acid PET studies showed paradoxically higher tracer uptakes in *IDH*-mutant gliomas, especially with oligodendroglial components, compared with counterpart *IDH1*-wildtype tumours [8–10]. These results suggest that amino acid PET studies are not fully consistent or accurate for differentiating glioma at diagnosis based on the 2016 WHO classification.

3′-deoxy-3′-[^18^F]fluorothymidine (FLT), a fluorinated thymidine analogue, has emerged as a promising PET tracer for evaluating tumour proliferating activity in various malignant tumours. FLT allows the direct measurement of cellular thymidine kinase-1 (TK1), which has been reported to be proportional to the proliferation activity of a tumour [11, 12]. As FLT uptake in normal brain tissue is very low, FLT-PET provides a low-background brain image and thus is considered useful for imaging brain tumours. FLT-PET has been found to be useful for assessing the proliferative activity of gliomas in vivo [13–15]. However, several studies have shown that the major portion of FLT uptake is dependent on the influx through the disrupted blood-brain barrier (BBB) and non-enhancing tumours with an intact BBB showed limited transport of FLT [15–18].

Previously, we reported that FLT-PET was superior to MET-PET in non-invasive tumour grading and assessment of proliferative activity in gliomas of different grades [13]. The study was conducted based on the 2007 WHO classification, and no study has evaluated the usefulness of FLT-PET for glioma differentiation based on the revised 2016 WHO classification. In the present study, we retrospectively analysed MET-PET/CT and FLT-PET/CT in newly diagnosed gliomas for differentiating glioma according to the 2016 WHO classification especially in relation to *IDH1* mutation status.

## Materials and methods

### Patients

From April 2009 to March 2019, 81 patients with newly diagnosed supratentorial glioma (36 men and 45 women; median age 62 years, range 21–86 years) were retrospectively enrolled in this study. All patients underwent routine magnetic resonance imaging (MRI) examinations including gadolinium (Gd)-enhanced T1-weighted MRI usually on the day before surgery. Patients underwent PET/CT studies with MET and FLT within a short period before surgery (median 14 days). The mean interval between the 2 PET examinations was 3.0 ± 4.3 days. Histopathology including immunohistochemistry (IHC) was performed on tissue specimens obtained by biopsy or resection using a multimodal navigation system (StealthStation, Medtronic-Sofamor Danek) by integrating PET/CT images with anatomical MR imagines. All gliomas were classified or reclassified using the 2016 WHO classification. The presence of *IDH1* mutation was assessed by IHC to detect *IDH1* R132H (codon 132 of the *IDH1* gene) protein expression. *IDH1* sequencing was performed when the IHC studies were negative. The presence or absence of 1p/19q codeletion was determined by fluorescence in situ hybridization analysis. The 1p/19q codeletion status is another important genetic alteration in the 2016 WHO classification. The presence of both the IHD1 mutation and 1p/19q codeletion characterizes oligodendrogliomas, whereas the presence of the *IDH1* mutation without 1p/19q codeletion is indicative of astrocytomas [1].

The patients’ characteristics, including histopathological and genetic diagnoses based on the 2016 WHO classification were summarized in Table 1. Six of 9 diffuse astrocytomas (DAs), 2 of 3 oligodendrogliomas (ODs), 3 of 14 anaplastic astrocytomas *(*AAs), 2 of 10 anaplastic oligodendrogliomas (AOs) and none of 45 *g*lioblastoma multiforme (GBMs), in total, 13 of 81 tumours (16%), were non-enhancing with Gd enhancement.

**Table 1:** Summary of 81 patients

Characteristics	Value
Age (years), median (range)	62 (21–86)
Sex, *n* (%)	
Male	36 (44.4)
Female	45 (55.6)
WHO 2016 grade, *n* (%)	
II	12 (14.8)
III	24 (29.6)
IV	45 (55.6)
Histology, *n* (%)	
Astrocytoma	68
DA	9 (13.2)
AA	14 (20.6)
GBM	45 (66.2)
Oligodendroglioma	13
OD	3 (23.1)
AO	10 (76.9)
IDH1 mutation, *n* (%)	
Mutant	29 (35.8)
Wildtype	52 (64.2)

### MET and FLT synthesis and PET acquisition

We routinely examine MET- and FLT-PET/CT in patients with suspected glioma. The clinical use of MET and FLT as a PET/CT tracer was approved by the Kagawa University Faculty of Medicine Human Subjects Ethical Committee, and written informed consent was obtained from all patients before PET/CT examinations.

MET and FLT were produced using an HM-18 cyclotron (Sumitomo Heavy Industries Ltd., Tokyo, Japan). MET and FLT were synthesized using the methods previously described [13] and radiochemical purity of the produced MET and FLT were > 99% and > 95%, respectively. PET examinations were performed using a Biograph mCT64 PET/CT scanner (Siemens/CTI, Knoxville, TN, USA). The imaging systems enabled simultaneous acquisition of 74 transverses per field of view (FOV), with an intersection spacing of 3 mm, for a total axial FOV of 21.6 cm. The in-plane transverse reconstructed resolution was 4.3 mm full width at half maximum (FWHM) in the brain FOV. No special dietary instructions were given to the patients before the PET/CT examinations. Images were acquired with patients resting in the supine position with their eyes closed. CT data were acquired first (tube rotation time, 0.6 s per revolution; 120 kV; 192 mAs; reconstructed slice thickness of 3 mm) and used for attenuation correction and anatomical localization of the tumours. For MET-PET/CT, a dose of 182–448 MBq (mean, 316 ± 55.6 MBq) of MET was injected intravenously and regional emission images were obtained for 5 min, beginning 10 min after MET administration. For FLT-PET/CT, a dose of 141–398 MBq (mean, 302 ± 45.2 MBq) of FLT was injected intravenously and regional emission images were obtained for 15 min, beginning 60 min after FLT administration. Image reconstruction was performed using ordered subset expectation maximization (OSEM) with time of flight (TOF) and point spread function (PSF). The reconstruction parameters were 2 iterations and 21 subsets. The FWHM of the Gaussian filter was 3 mm.

### Image analysis

MET and FLT uptakes in brain lesions were semiquantitatively assessed by evaluating the standardized uptake value (SUV). A region of interest (ROI) was set manually by a nuclear medicine physician (YY or YN) who was blind to pathological information on the hottest area of each lesion or its centre located by MRI with fluid-attenuated inversion recover (FLAIR) sequences, if increased MET and FLT uptake was absent. The maximum value of SUV (SUVmax) was regarded as the representative value of each tumour. To calculate the tumour-to-normal tissue count density (T/N) ratios, the ROI was set on the normal brain parenchyma (usually contralateral to normal cortex), and the mean value of SUV (SUVmean) was calculated. The T/N ratio was determined by dividing the SUVmax of the tumour by the SUVmean of the normal brain tissue.

### Statistical analysis

The Mann-Whitney *U* test was used to compare the T/N ratios of MET and FLT in relation to *IDH1* mutation and 1p/19q codeletion status. For multiple comparison, the Kruskal-Wallis test was used to compare the T/N ratios of MET and FLT in relation to WHO grade and histopathological classification on pathology. The Steel-Dwass method was used in post hoc analysis. Receiver operating characteristic (ROC) analysis was performed to compare the diagnostic usefulness of T/N ratios of MET and FLT in predicting *IDH1* mutation status. We determined the cutoff value at which the sum of sensitivity and specificity (Youden’s index) was the highest. All statistical analyses were performed using EZR version 1.40 (Saitama Medical Center, Jichi Medical University, Saitama, Japan). A *P* value less than 0.05 was considered statistically significant.

## Results

### Tracer uptake in relation to histological grade

The median MET T/N ratios in grade II (*n* = 12), grade III (*n* = 24) and grade IV (*n* = 45) gliomas were 2.51 (interquartile range [IQR] 1.82–3.13), 5.03 (3.58–6.39) and 5.99 (4.64–7.39), respectively (Table 2). There were significant differences in the median MET T/N ratios between grade II and III gliomas (*P* < 0.001) and grade II and IV gliomas (*P* < 0.001), but not between grade III and IV gliomas. The median FLT T/N ratios in grade II, grade III and grade IV gliomas were 2.24 (IQR 1.69–2.62), 6.25 (4.59–8.69) and 15.24 (11.11–20.49), respectively. There were significant differences in the median FLT T/N ratios between grade II and III gliomas (*P* < 0.001) and grade III and IV gliomas (*P* < 0.001).

**Table 2:** SUVmax and T/N ratio of gliomas

Characteristic	MET		FLT	
	SUVmax, median (IQR)	T/N ratio, median (IQR)	SUVmax, median (IQR)	T/N ratio, median (IQR)
Grade				
II (*n* = 12)	2.99 (1.94–4.23)	2.51 (1.86–3.13)	0.49 (0.30–0.69)	2.24 (1.69–2.62)
III (*n* = 24)	5.92 (4.65–6.52)	5.03 (3.58–6.39)	1.29 (0.90–1.49)	6.25 (4.59–8.69)
IV (*n* = 45)	6.21 (4.45–7.66)	5.99 (4.64–7.39)	2.77 (1.92–3.29)	15.24 (11.11–20.49)
IDH status				
Mutant (*n* = 29)	4.83 (3.04–6.37)	3.6 (2.87–5.59)	0.93 (0.47–1.44)	4.18 (2.28–6.39)
Wildtype (*n* = 52)	6.11 (4.63–7.56)	5.91 (4.54–7.35)	2.59 (1.61–3.24)	14.7 (8.98–20.39)
Histology and subtype				
DA (*n* = 9)	2.77 (1.86–3.69)	2.03 (1.83–2.97)	0.46 (0.23–0.57)	2.35 (1.69–2.60)
IDH-mut (*n* = 8)	2.76 (1.75–4.01)	2.45 (1.85–3.26)	0.45 (0.23–0.59)	2.07 (1.69–2.62)
IDH-wt (*n* = 1)	2.85	1.83	0.57	2.48
OD (*n* = 3)	3.68 (3.31–4.29)	2.86 (2.51–3.23)	0.59 (0.49–0.73)	2.14 (1.91–3.42)
AA (*n* = 14)	5.22 (4.06–5.84)	4.65 (3.25–5.99)	1.37 (0.89–1.47)	7.16 (4.77–9.41)
IDH-mut (*n* = 6)	4.50 (3.14–5.67)	3.28 (2.99–5.19)	0.99 (0.68–1.35)	4.9 (3.48–6.98)
IDH-wt (*n* = 8)	5.76 (5.26–5.83)	5.41 (4.11–6.20)	1.66 (0.90–2.36)	8.1 (6.58–14.53)
AO (*n* = 10)	6.91 (5.14–7.79)	5.07 (4.72–7.21)	1.18 (0.97–1.49)	5.82 (4.26–6.79)
GBM (*n* = 45)	6.21 (4.45–7.66)	5.99 (4.64–7.39)	2.77 (1.92–3.29)	15.24 (11.11–20.46)
IDH-mut (*n* = 2)	5.44 (4.64–6.24)	5.6 (5.59–5.60)	1.90 (1.74–2.06)	14.58 (14.47–14.69)
IDH-wt (*n* = 43)	6.25 (4.58–7.71)	6.19 (4.63–7.41)	2.81 (1.95–3.32)	15.65 (10.35–20.77)

### Tracer uptake in relation to histopathological classification

**Fig. 1:**
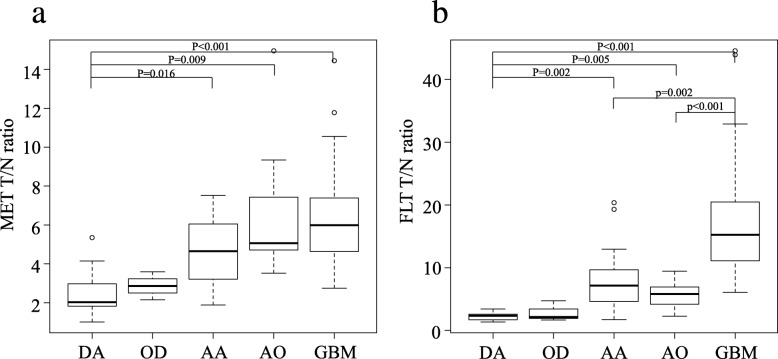
Box plots showing the T/N ratios of MET (**a**) and FLT (**b**) in relation to histopathological classification. OD was omitted from the statistical analysis because of the small number of cases (*n* = 3). There were significant differences in the median MET T/N ratios between DA and AA (*P* = 0.016), DA and AO (*P* = 0.009), and DA and GBM (*P* < 0.001) (**a**). There were significant differences in the median FLT T/N ratios between DA and AA (*P* = 0.002), DA and AO (*P* = 0.005), DA and GBM (*P* < 0.001), AA and GBM (*P* = 0.002), and AO and GBM (*P* < 0.001) (**b**)

The median MET T/N ratios in DA (*n* = 9), OD (*n* = 3), AA (*n* = 14), AO (*n* = 10) and GBM (*n* = 45) were 2.03 (IQR 1.83–2.97), 2.86 (2.51–3.23), 4.65 (3.25–5.99), 5.07 (4.72–7.21) and 5.99 (4.64–7.39), respectively (Table 2). OD was omitted from the statistical analysis because of the small number of cases. There were significant differences of the median MET T/N ratios between DA and AA (*P* = 0.016), DA and AO (*P* = 0.009), and DA and GBM (*P* < 0.001; Fig. 1a). The median FLT T/N ratios in DA, OD, AA, AO and GBM were 2.35 (IQR 1.69–2.60), 2.14 (1.91–3.42), 7.16 (4.77–9.41), 5.82 (4.26–6.79) and 15.24 (11.11–20.46), respectively. There were significant differences in the median FLT T/N ratios between DA and AA (*P* = 0.002), DA and AO (*P* = 0.005), DA and GBM (*P* < 0.001), AA and GBM (*P* = 0.002), and AO and GBM (*P* < 0.001; Fig. 1b).

### Tracer uptake in relation to *IDH1* mutation status

**Fig. 2:**
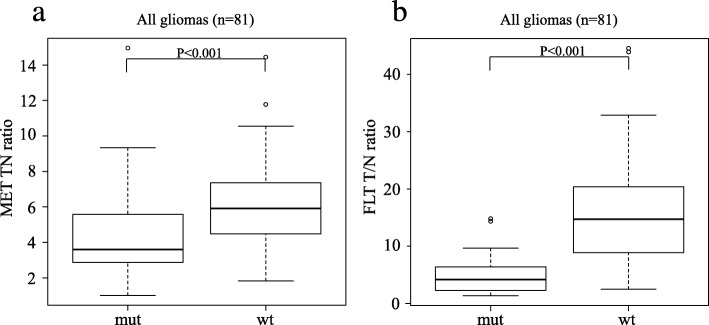
Box plots showing the T/N ratios of MET (**a**) and FLT (**b**) in relation to *IDH1* mutation status in all gliomas (*n* = 81). The tracer uptake in *IDH1*-wildtype (wt) tumours was significantly higher than that in *IDH1*-mutant (mut) tumours both in MET-PET (*P* < 0.001) and FLT-PET (*P* < 0.001)

**Fig. 3:**
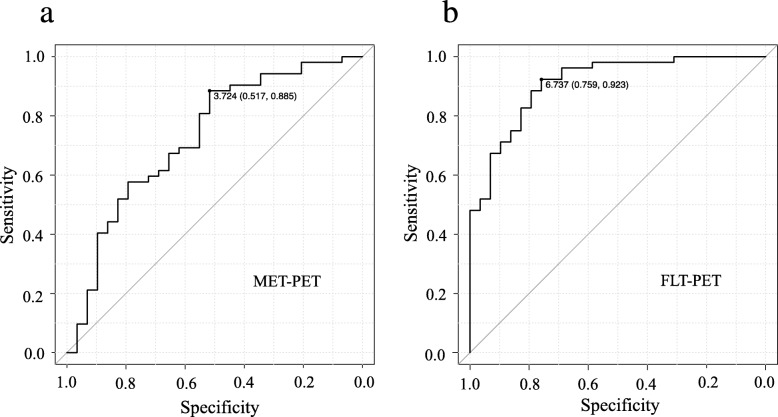
ROC curve of MET (**a**) and FLT (**b**) for differentiating *IDH1*-wildtype from *IDH1*-mutant tumours in all gliomas (*n* = 81). The area under the curve (AUC) of the FLT T/N ratio (AUC: 0.911, 95% CI 0.847–0.975) was significantly larger than that of the MET T/N ratio (AUC: 0.727, 95% CI 0.607–0.847)

In all gliomas, the median MET T/N ratios in *IDH1*-mutant and *IDH1*-wildtype tumours were 3.6 (IQR 2.84–5.59) and 5.91 (4.57–7.35), respectively. The median MET T/N ratio in *IDH1*-wildtype tumours was significantly higher than that in *IDH1*-mutant tumours (*P* < 0.001; Fig. 2a). The median FLT T/N ratio in *IDH1*-mutant and *IDH1*-wildtype gliomas were 4.18 (IQR 2.28–6.39) and 14.7 (8.98–20.38), respectively. Again, the median FLT T/N ratio in *IDH1*-wildtype tumours was significantly higher than that in *IDH1*-mutant tumours (*P* < 0.001; Fig. 2b). There was a significant overlap of MET T/N ratios between *IDH1*-mutant and *IDH1*-wildtype tumours. On the other hand, the overlap of FLT T/N ratios between *IDH1*-mutant and *IDH1*-wildtype tumours was small. ROC analysis for differentiating *IDH1*-mutant tumours from *IDH1*-wildtype tumours showed that the area under the curve (AUC) of the FLT T/N ratio (AUC 0.911, 95% CI 0.847–0.975; Fig. 3b) was significantly larger than that of the MET T/N ratio (AUC 0.727, 95% CI 0.607–0.847; Fig. 3a) (*P* < 0.01). When the cutoff value of the FLT T/N ratio in the ROC curve was set at 6.74, the sensitivity for the differential diagnosis was 92.3%, and the specificity was 75.9% (Fig. 3b). On the other hand, the sensitivity for the differential diagnosis was 88.5%, and the specificity was 51.7% when the cutoff value of the MET T/N ratio was set at 3.72 (Fig. 3a).

**Fig. 4:**
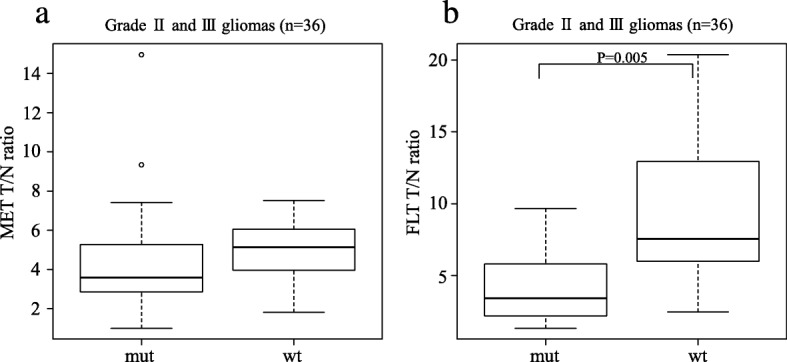
Box plots showing the T/N ratios of MET (**a**) and FLT (**b**) in relation to *IDH1* mutation status in grade II and III gliomas (*n* = 36). There was no significant difference in the median MET T/N ratios between *IDH1*-mutant (mut) and *IDH1*-wildtype (wt) tumours (**a**). The median FLT T/N ratio in *IDH1*-wildtype tumours was significantly higher than that in *IDH1*-mutant tumours (*P* = 0.005) (**b**)

When analysing the uptake values in 36 grade II and III gliomas separately, the median MET T/N ratios in *IDH1*-mutant and *IDH1*-wildtype tumours were 3.59 (IQR 2.87–5.28) and 5.14 (3.97–6.06), respectively. There was no significant difference in the median MET T/N ratios between *IDH1*-mutant and *IDH1*-wildtype tumours (Fig. 4a). In the same population, the median FLT T/N ratios in *IDH1*-mutant and *IDH1*-wildtype tumours were 3.43 (IQR 2.20–5.82) and 7.56 (6.00–12.94), respectively. The median FLT T/N ratio in *IDH1*-wildtype tumours was significantly higher than that in *IDH1*-mutant tumours (*P* = 0.005; Fig. 4b).

**Fig. 5:**
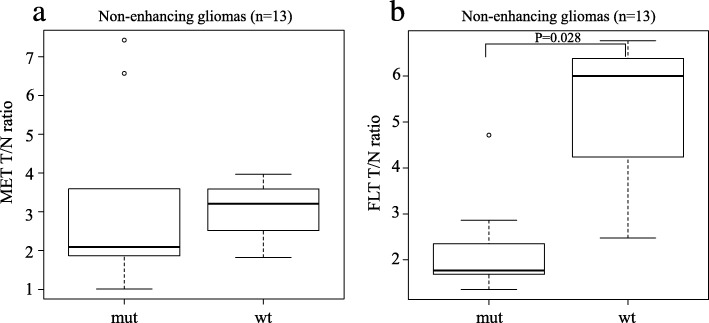
Box plots showing the T/N ratios of MET (**a**) and FLT (**b**) in relation to *IDH1* mutation status in non-enhancing gliomas (*n* = 13). There was no significant difference in the median MET T/N ratios between *IDH1*-mutant (mut) and *IDH1*-wildtype (wt) tumours (**a**). The median FLT T/N ratio in *IDH1*-wildtype tumours was significantly higher than that in *IDH1*-mutant tumours (*P* = 0.028) (**b**)

When analysing the uptake values in 13 non-enhancing tumours (9 grade II and 4 grade III) separately, the median MET T/N ratios in *IDH1*-mutant (*n* = 10) and *IDH1*-wildtype (*n* = 3) tumours were 2.09 (IQR 1.87–3.44) and 3.21 (2.52–3.59), respectively. There was no significant difference in the median MET T/N ratios between *IDH1*-mutant and *IDH1*-wildtype tumours (Fig. 5b). In the same population, the median FLT T/N ratios in *IDH1*-mutant and *IDH1*-wildtype tumours were 1.77 (IQR 1.68–2.33) and 6.00 (4.23–6.38), respectively. The median FLT T/N ratio in *IDH1*-wildtype tumours was significantly higher than that in *IDH1*-mutant tumours (*P* = 0.028; Fig. 5b).

### Tracer uptake in relation to histological grade in IDH1-mutant and wildtype tumours

**Fig. 6:**
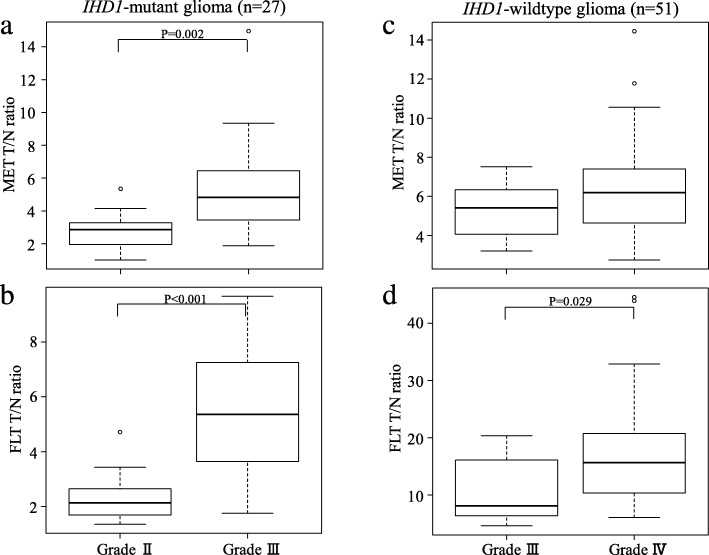
Box plots showing the T/N ratios of MET and FLT in relation to glioma grading in *IDH1*-mutant tumours (**a**, **b**) and in *IDH1*-wildtype tumours (**c**, **d**). In *IDH1*-mutant tumours, the tracer uptake in grade III gliomas was significantly higher than that in grade II gliomas both in MET-PET (*P* = 0.002) (**a**) and FLT-PET (*P* < 0.001) (**b**). In *IDH1*-wildtype tumours, there was no significant difference in the median MET T/N ratios between grade III and IV gliomas (**c**). The median FLT T/N ratio in grade IV gliomas was significantly higher than that in grade III gliomas (*P* = 0.029) (**d**)

In the present study, there were only 2 *IDH1*-mutant GBMs among the 29 *IDH1*-mutant tumours. We therefore excluded the 2 *IDH1*-mutant GBMs from the analysis. In the remaining 27 *IHD1*-mutant tumours, the median MET T/N ratios were 2.86 (IQR 1.95–3.28) and 4.83 (3.48–6.39) for the grade II (*n* = 11) and III (*n* = 16) gliomas, respectively. The median MET T/N ratio in the grade III gliomas was significantly higher than that in the grade II gliomas (*P* = 0.002; Fig. 6a). In the same population, the median FLT T/N ratios in the grade II and III gliomas were 2.14 (IQR 1.69–2.64) and 5.36 (3.90–7.09), respectively. Again, the median FLT T/N ratio in the grade III gliomas was significantly higher than that in the grade II gliomas (*P* < 0.001; Fig. 6b).

In the 52 *IDH1*-wildtype tumours, there was only 1 *IDH1*-wildtype DA. We therefore excluded the DA from the analysis. In the remaining 51 *IHD1*-wildtype tumours, the median MET T/N ratios were 5.41 (IQR 4.11–6.20) and 6.19 (4.63–7.41) for the grade III (*n* = 8) and IV (*n* = 43) gliomas, respectively. There was no significant difference of the median MET T/N ratios between grade III and IV gliomas (Fig. 6c). In the same population, the median FLT T/N ratios in the grade III and IV gliomas were 8.10 (IQR 6.58–14.53) and 15.65 (10.35–20.77), respectively. The median FLT T/N ratio in the grade IV gliomas was significantly higher than that in the grade III gliomas (*P* = 0.029; Fig. 6d).

## Discussion

*IDH1* mutation status is the genetic alteration with the most significant impact on the updated 2016 edition of the WHO Classification of Tumours of the CNS [1, 2]. Patients with *IDH1*-mutant astrocytomas have a better overall prognosis compared with those with *IDH1*-wildtype astrocytomas, even after controlling for histologic grade [1, 3]. Given their distinct molecular origins, the optimal treatment strategies for *IDH1*-mutant versus *IDH1*-wildtype tumours should be different. Recently, several studies have reported that response and benefit to treatment differ depending on the *IHD1* mutation status [19–21]. Based on these studies, preoperative prediction of genotypes, especially the *IDH1* mutation status, is essential to planning tailored treatment strategies including surgical resection and postoperative adjuvant therapy. This is especially the case with WHO grade II and III gliomas [20]. On the other hand, the great majority of WHO grade IV astrocytomas falls into the *IDH1*-wildtype category (more than 90% of cases) [1], which corresponds most frequently to the clinically defined primary GBM resulting in poor prognosis even with intensive treatments.

Based on the 2016 WHO classification, MET-PET studies in patients with newly diagnosed gliomas have reported a correlation between MET uptake and *IDH1* mutation status, showing that *IDH1*-wildtype gliomas had significantly higher MET uptake than *IDH1*-mutant gliomas [6–8]. In the present study, the MET uptakes in *IDH1*-wildtype tumours were also significantly higher than that in *IDH1*-mutant tumours in all gliomas, but this was not the case among grade II and III gliomas. Although the sensitivity for the differential diagnosis was high (88.5 %), the specificity was low (51.7 %) using MET-PET/CT. Kim et al. reported that among grade II and III gliomas, *IDH1*-mutant and 1p/19q-codeleted oligodendrogliomas were more likely to exhibit higher MET uptake, even paradoxically high, compared with the counterpart *IDH1*-wildtype astrocytomas [8]. They cautioned that MET uptake for glioma grading according to the 2016 WHO classification was more consistent and accurate for *IDH1*-wildtype tumours than for *IDH1*-mutant tumours. Previous studies have shown that MET uptake in gliomas with an oligodendroglial component is higher than in astrocytomas, even in low-grade gliomas [13, 22, 23]. High MET uptakes in *IDH1*-mutant oligodendrogliomas may cause low specificity for the differential diagnosis. In fact, AOs (median 5.07, IQR 4.72–7.21) showed increased MET uptake as high as those in GBMs (median 6.19, IQR 4.63–7.41), in which most of them were *IDH1*-wild type tumours in the present study. Several amino acid PET studies showed paradoxically higher tracer uptakes in *IDH-mutant gliomas* and the inability to predict the *IDH1* mutation status among grade II and III gliomas [9, 10]. These results suggest that the use of amino acid PET for differentiating glioma at diagnosis may be useful but is subject to controversy. Recently, several studies have shown the usefulness of amino acid PET with dynamic analysis for glioma differentiation based on the 2016 WHO classification [10, 24].

3′-Deoxy-3′-[^18^F]fluorothymidine (FLT), a fluorinated thymidine analogue, has emerged as a promising PET tracer for evaluating tumour proliferating activity in various brain tumours. FLT is phosphorylated by TK1, a principle enzyme in the salvage pathway of DNA synthesis, and trapped inside cells. Phosphorylated FLT is resistant to degradation and suitable for imaging with PET. The application of FLT phosphorylation as a marker of cell proliferation is based on the assumption that cellular FLT trapping is a representation of thymidine incorporation into DNA [11, 12]. FLT-PET has been found useful for non-invasive grading and assessment of proliferative activity especially in newly diagnosed gliomas [13–15]. Moreover, FLT-PET can provide valuable information regarding treatment response and patient prognosis and regarding the recognition of tumour recurrence in gliomas [25, 26]. In our previous study, FLT-PET was found to be likely superior to MET-PET in tumour grading and assessment of proliferative activity in newly diagnosed gliomas of different grades [13]. In the present study, MET-PET/CT could only discriminate DA from other histological types of gliomas, i.e. DA and AA, DA and AO, and DA and GBM. On the other hand, FLT-PET/CT can distinguish all different types of gliomas except AA and AO based on the 2016 WHO classification. Moreover, both PET tracers could distinguish grade II gliomas from higher-grade gliomas, but the differentiation between grade III and IV gliomas, two groups with different prognosis and management [27], was able to achieve only with FLT-PET/CT.

This is the first study to demonstrate a significant correlation between FLT uptake values and *IDH1* mutation status in newly diagnosed gliomas. FLT uptakes in *IDH1*-wildtype tumours were significantly higher than those in *IDH1*-mutant tumours. ROC analysis showed that FLT-PET/CT can distinguish the *IDH1* mutation status more accurately than MET-PET/CT. Histologically, the prognostic differences between DA and AA were significant [28]. However, recent studies have shown that the prognostic difference between *IHD*-mutant DAs and *IHD*-mutant AAs are not as marked [29], suggesting that *IDH* status might be more important than tumour grade. In the present study, more than 50% of the gliomas were GBMs, and almost all GBMs were *IDH1*-wildtype (43/45 cases). PET tracer uptake in GBMs is usually higher than that in other grades of gliomas, and this may influence the results in relation to *IDH1* mutation status. Therefore, we conducted further examination of the uptake values in 36 grade II and III gliomas. It should be noted that FLT-PET/CT was able to distinguish the *IDH1*-mutant tumours from wildtype tumours not only in all gliomas, but also in this specific population, but this was not the case for MET-PET/CT. Although the sensitivity for differential diagnosis of *IDH1* mutation status in the ROC analysis was high (92.3%) with FLT-PET, the specificity was not satisfactory (75.9%). This number means that one in 4 gliomas with low FLT uptake below the cutoff value (T/N ratio of 6.74) was an *IHD1*-wildtype tumour that could harbour an aggressive nature in the tumour. Our previous study showed that FLT uptake in tumours without an observed contrast enhancement effect on MR images was low even if the tumours were high-grade gliomas [15]. Later studies, including from our laboratory, have shown that the major portion of FLT uptake is due to increased transport and influx through the disrupted blood-brain barrier (BBB) [16, 17]. Non-enhancing tumours with an intact BBB showed limited transport of FLT, and tumour malignancy and cell proliferation activity cannot be adequately assessed by FLT-PET [18]. In the present study, FLT-PET/CT was able to distinguish the *IDH1*-mutant tumours from wildtype tumours statistically. However, the number of non-enhancing tumours was limited (*n* = 13), especially the *IDH1*-wildtype tumours (*n* = 3) and the result was not conclusive. The issue regarding the usefulness of FLT-PET/CT for differentiating *IDH1* mutation status in non-enhancing tumours should be addressed with more cases in the future.

The present study had several limitations. First, there was selection bias with respect to the relatively small number of patients with low-grade glioma. Patients with low tracer uptakes were suspected of having low-grade gliomas and considered less likely candidates for surgery, especially when they had few or no symptoms. Second, patients with *IHD1*-wildtype DA (*n* = 1) and *IHD1*-mutant GBMs (*n* = 2) were excluded from the statistical analysis because of the small number of cases. We only compared the tracer uptakes in grade II and III gliomas with *IDH1* mutation and grade III and IV gliomas without *IDH1* mutation. Incidences of *IHD1*-mutant GBM (less than 10%) and *IHD1*-wildtype DA are rare in the general population [1, 30]. Third, we have not evaluated the tracer uptakes in relation to *IDH1* mutation status within the same WHO malignancy grade due to too few datasets available for a meaningful analysis. Patients harbouring the *IDH1* mutation had a longer overall survival than those without mutation within AA as well as GBM [31]. Finally, in addition to the retrospective nature of the present study, no follow-up data were evaluated in the present study, and the findings need to be analysed in relation to prognosis with a long flow-up period.

### Conclusions

*IDH1*-wildtype tumours showed significantly higher FLT uptake than *IDH1*-mutant tumours. The accuracy for differentiating *IDH1* mutation status with FLT-PET/CT was higher than with MET-PET/CT. FLT-PET/CT was able to distinguish the *IDH1*-mutant tumours from wildtype tumours not only in all gliomas but also in grade II and III gliomas. Moreover, only FLT-PET/CT was able to distinguish both between grade III and IV gliomas in *IDH1*-wildtype tumours and grade II and III gliomas in *IDH1*-mutant tumours FLT-PET/CT can improve glioma differentiation based on the 2016 WHO classification in newly diagnosed gliomas, but caution must be paid for tumours without contrast enhancement and further studies should be conducted with more cases.

## Data Availability

Due to the sensitive nature of human participant information, all datasets used during the current study are not publicly open but available upon reasonable request by contacting the corresponding author.

## Abbreviations

AA: Anaplastic astrocytoma
AO: Anaplastic oligodendroglioma
*AUC*: Area under the curve
CT: Computed tomography
*DA*: Diffuse astrocytoma
DNA: Deoxyribonucleic acid
EZR: Easy R
FLT: ^18^F-fluorothymidine
FOV: Field of view
FWHM: Full width at half maximum
GBM: Glioblastoma multiforme
Gd: Gadolinium
IDH1: *Isocitrate dehydrogenase* 1
IHC: *Immunohistochemistry*
IQR: *Interquartile range*
MET: ^11^C-methionine
MRI: Magnetic resonance imaging
OD: Oligodendroglioma
OSEM: Ordered subset expectation maximization
PET: Positron emission tomography
PSF: Point spread function
ROC: Receiver operating characteristic
ROI: Region of interest
SUV: Standardized uptake value
SUVmax: Maximum value of SUV
SUVmean: Mean value of SUV
T/N: Tumour-to-normal
TK1: Thymidine kinase-1
TOF: Time of flight
WHO: World Health Organization
